# Heterologous Protein Expression Is Enhanced by Harmonizing the Codon Usage Frequencies of the Target Gene with those of the Expression Host

**DOI:** 10.1371/journal.pone.0002189

**Published:** 2008-05-14

**Authors:** Evelina Angov, Collette J. Hillier, Randall L. Kincaid, Jeffrey A. Lyon

**Affiliations:** 1 Molecular Parasitology, Division of Malaria Vaccine Development, Walter Reed Army Institute of Research, Silver Spring, Maryland, United States of America; 2 Veritas, Inc., Rockville, Maryland, United States of America; Baylor College of Medicine, United States of America

## Abstract

Synonymous codon replacement can change protein structure and function, indicating that protein structure depends on DNA sequence. During heterologous protein expression, low expression or formation of insoluble aggregates may be attributable to differences in synonymous codon usage between expression and natural hosts. This discordance may be particularly important during translation of the domain boundaries (link/end segments) that separate elements of higher ordered structure. Within such regions, ribosomal progression slows as the ribosome encounters clusters of infrequently used codons that preferentially encode a subset of amino acids. To replicate the modulation of such localized translation rates during heterologous expression, we used known relationships between codon usage frequencies and secondary protein structure to develop an algorithm (“codon harmonization”) for identifying regions of slowly translated mRNA that are putatively associated with link/end segments. It then recommends synonymous replacement codons having usage frequencies in the heterologous expression host that are less than or equal to the usage frequencies of native codons in the native expression host. For protein regions other than these putative link/end segments, it recommends synonymous substitutions with codons having usage frequencies matched as nearly as possible to the native expression system. Previous application of this algorithm facilitated *E. coli* expression, manufacture and testing of two *Plasmodium falciparum* vaccine candidates. Here we describe the algorithm in detail and apply it to *E. coli* expression of three additional *P. falciparum* proteins. Expression of the “recoded” genes exceeded that of the native genes by 4- to 1,000-fold, representing levels suitable for vaccine manufacture. The proteins were soluble and reacted with a variety of functional conformation-specific mAbs suggesting that they were folded properly and had assumed native conformation. Codon harmonization may further provide a general strategy for improving the expression of soluble functional proteins during heterologous expression in hosts other than *E. coli*.

## Introduction

Changes to protein structure and function that occur after synonymous codon replacement indicate that protein structure is DNA sequence dependent. Synonymous codon substitutions that change codon usage frequencies from infrequent to frequent usage in regions of slow mRNA translation can deleteriously affect enzyme activity [1]. Conversely, synonymous substitutions that introduce rare codons into regions predicted to contain high frequency codons show altered substrate specificities [2]. Thus, contrary to conventional thinking, synonymous codon substitutions may not always be silent; changing codon usage frequency affects protein structure and function, and the frequency with which codons are used imparts vital information for the development of secondary and tertiary protein structure.

Species-specific disparities in codon usage are frequently cited as the cause for failures in recombinant gene expression by heterologous expression hosts. Such failures include lack of expression, or the expression of protein that is non-functional or insoluble, or protein that is truncated owing to proteolysis or premature termination of translation [3]–[5]. All but the last of these failures are attributable to misfolded protein. In *Escherichia coli* as well as eukaryotic species, nascent proteins fold co-translationally within the ribosomal tunnel, which is both a protective environment within which secondary structure begins to form [6]–[8], and a dynamic environment that influences nascent protein structure [9]–[12]. Within the ribosomal tunnel, subtle variations in the rate of mRNA translation may play a key role in developing secondary structure in the nascent protein. Translation is not a steady state process, rather it occurs in pulses, as can be observed from ribosomal pausing [13] and even ribosome stacking, on specific stretches of mRNA [14]; these temporal changes in translational rate have been shown to depend on relative tRNA levels [15].

tRNA isoacceptor abundance and isoacceptor usage frequencies are directly related for naturally occurring proteins from *E. coli* as well as from other organisms [16]–[19], and there is evidence that protein secondary structure is related to tRNA usage frequencies [20], although this concept is controversial [21]. Comparative analysis of *E. coli* gene sequences and their respective protein structures show that amino acid sequences encoded by more frequently used codons are associated with highly ordered structural elements such as alpha helices, while sequences containing clusters of less frequently used codons tend to be associated with the protein domain boundaries (link/end segments) that separate such elements [22]. That analysis also showed that the link/end segments tend to be populated with amino acids that have bulky hydrophobic side chains or side chains that can hydrogen bond to the peptide backbone. When such residues appear in link/end segments, they tend to be encoded by infrequently-used codons. Therefore, the positioning of clusters of relatively high and low abundance codons on mRNA transcripts may be a purposeful rather than a random occurrence [23]. The idea that link/end segments, which separate elements of higher order protein structure, are encoded by clusters of low-usage frequency codons leads to the hypothesis that slow translational progression (i.e., “pausing”) through such regions of mRNA would allow the preceding nascent structural element to fold, at least partially, within the environment of the ribosomal tunnel prior to initiation of synthesis of the next structural element. Such a temporal control mechanism would minimize the interaction between partially folded nascent polypeptides in the cytosol, an event which can lead to degradation, or aggregation and precipitation.

Based on these concepts-that protein synthesis and folding in *E. coli* is co-translational and that nucleotide sequence-dependent modulation of translation kinetics might influence nascent polypeptide folding-we developed a strategy to “recode” target gene sequences for heterologous expression in *E. coli* by substituting the native codons with synonymous ones having the same or similar usage frequencies in the expression host. In this approach, termed “codon harmonization”, synonymous codons from *E. coli* were selected that match as closely as possible the codon usage frequency used in the native gene, unless empirical structure calculations show that the codons are associated with putative link/end segments and therefore should be translated slowly. Such regions were recoded by selecting the closest matching synonymous *E. coli* codon having a usage frequency equal to or less than that of its respective isoacceptor codon's usage frequency in the native gene's host.

Disharmony between codon usage patterns for *Plasmodium falciparum* malaria parasite target genes and *E. coli* has proven particularly challenging for heterologous expression in *E. coli* owing to the 80% AT bias in the structural genes from *P. falciparum* [24]. Previously, we showed that codon harmonization can overcome this challenge. FMP003[25], which was the result of a single codon change made in one predicted link/end segment, and LSA-NRC^H^ [26] for which the gene fragment was fully codon harmonized, were both manufactured under cGMP conditions and have progressed through pre-clinical or clinical evaluation as vaccine candidates. In those reports, we emphasized manufacture and evaluation of the products, but the details of the algorithm were not described sufficiently to allow replication. In this current work we explain the algorithm in detail, and apply it to the expression of the codon harmonized gene sequences for three additional malaria vaccine candidates, namely MSP1_42_ (FVO), MSP1_42_ (3D7) and MSP1_42_ (Camp). In all cases, both protein yield and protein solubility improved significantly. Protein yields were 3–12 mg purified protein/g wet cell paste. This increase in yield of soluble protein was critical, as it enabled these antigen candidates to be developed for clinical testing as malaria vaccines.

## Results

### Codon Harmonization Algorithm

**Figure 1:**
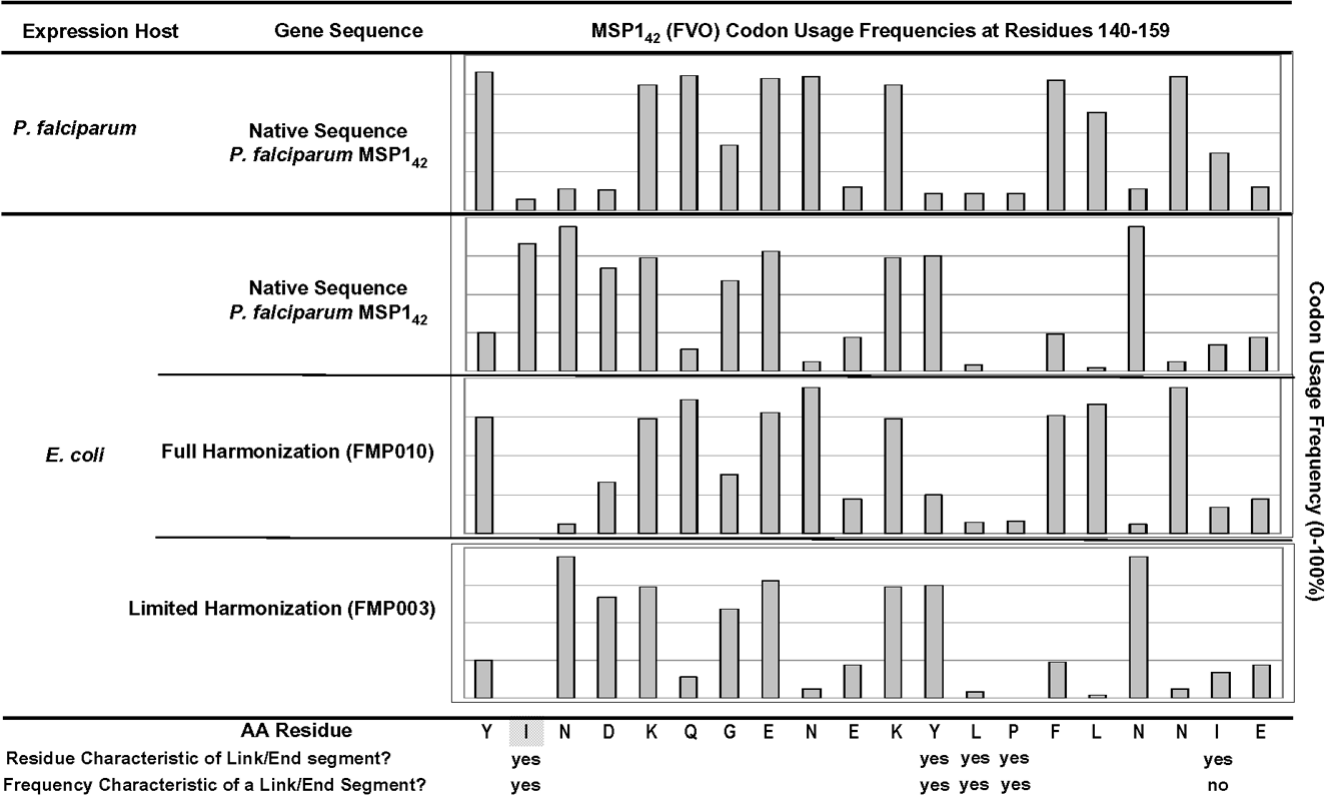
Comparison of Codon Usage Frequencies in the Native MSP1_42_ (FVO) and Recoded Sequences as a Function of Expression Host, for either native *P. falciparum* or *E. coli* expression. Amino acid segment of MSP1_42_ (FVO) sequence is shown (residue # 140–159) with the codon usage frequencies rounded to the nearest 10%. The shaded residue at I(141) was targeted for synonymous replacement in FMP003.

The identification of transcript regions that are likely to promote ribosomal pausing requires a means to predict such segments. Application of our algorithm shows a striking example of a region of the FVO allele of the MSP1_42_ gene fragment that was calculated to be a link/end segment (Fig. 1, top line). Nine of the 20 amino acids shown in this diagram are encoded by tRNA isoacceptor codons used 15% of the time or less in *P. falciparum*, where the summation of synonymous codons that could encode each residue equals 100%. Among these, four (I141, Y151, L152, P153) belong to the group of ten residues that tend to populate link/end segments of *E. coli* proteins having known structure and they were used at the rate of 11% of the time or less. I141 was included in the link/end group because it lies within 14 residues of the tricodon Y151-P153 and thus fits the criteria of the space parameter described by Thanaraj [20]. Although I158 satisfies the criterion of being among these ten residues and fits the space parameter, its isoacceptor usage frequency in the native expression host is greater than 25%; therefore this residue was not calculated to be part of the link/end segment. Introduction of the native *P. falciparum* MSP1_42_ gene sequence into *E. coli* resulted in a dramatic shift in codon usage frequencies within this region (compare first and second lines, respectively). Of the nine low frequency codons identified in the first line, five were shifted to higher usage frequencies (second line); I141 and Y151 are among these. In addition several codons, namely Q145, N148, Y154, L155, and N157, are used at high frequency in the native host (first line) but were used at low frequency in *E. coli* (second line). The usage frequency for the residue P153 (CCC) dropped from 11% in *P. falciparum* to 0.1% in *E. coli*. Following full codon harmonization of this segment for expression in *E. coli* (i.e., addressing the frequency of codon usage at each residue), codon usage patterns for heterologous expression (third line) were in good agreement with those of the gene in its native host (compare first and third line), e.g. with the usage rate for P153 increasing to 8% as compared to 11% in *P. falciparum*. A table showing the full procedure for application of the codon harmonization algorithm for this region of FVO strain MSP1_42_ has been included in the Supplementary Materials (Table S1). As described below, our initial studies evaluated the utility of this codon harmonization algorithm by focusing on this region of the gene because, of all of the putative regions of slowly translated mRNA that were identified, this was the most discordant in its codon usage pattern.

### Single Codon Application of Codon Harmonization

**Figure 2:**
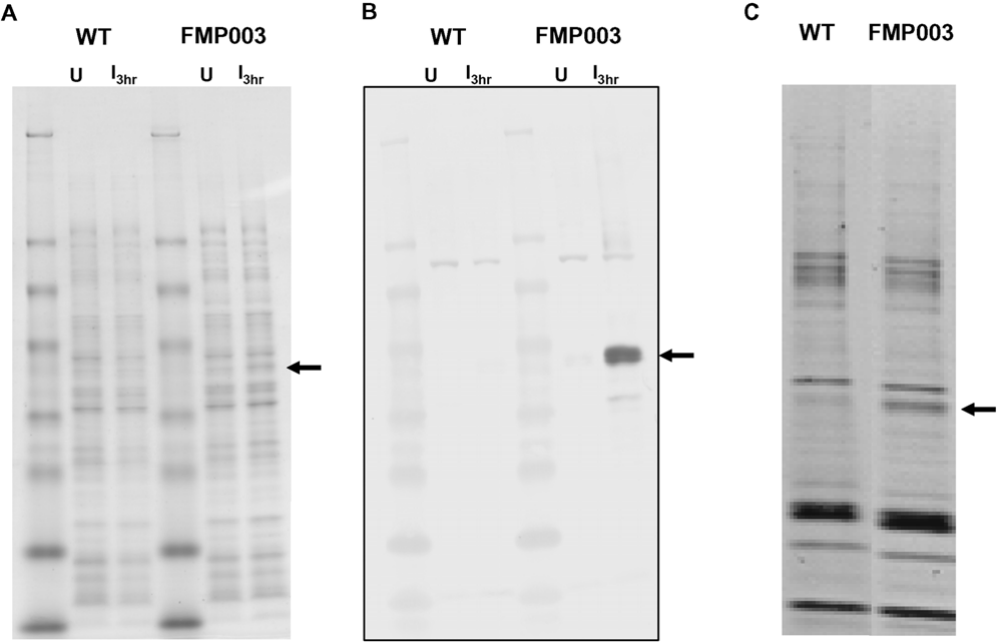
Comparison of MSP1_42_ (FVO) expression from plasmids encoding either wild type or the FMP003 gene by SDS-PAGE and Coomassie Blue staining or Western Blotting. Panel A, Coomassie Blue stained gels at the time of induction (U) or 3 hours post induction (I_ 3hr_); Panel B, Western blots at the time of induction (U) or 3 hours post induction (I_ 3hr_); Panel C, Coomassie Blue stained gels of protein following affinity purification on Ni^+2^-NTA chromatography. Arrows indicate migration of FMP003.

Initially, we wanted to test the value of this approach by focusing only on a limited section of sequence that should comprise a region of slow translation and whose usage frequency during heterologous expression would be strongly discordant. We determined that the region of the FVO allele of the MSP1_42_ gene fragment shown in Fig 1 met this requirement, and changed the codon for I141 (ATC→ATA) to test the strategy (Fig. 1, compare first and fourth line; the shaded box on the AA residue line identifies this residue). *E. coli* transformed with the altered gene expressed significantly more protein (designated FMP003) than was obtained from the wild type gene. Although, we could not detect expression of the FMP003 protein directly in cell lysates on gels stained with Coomassie Blue (Fig 2A), it was readily detected by western blotting whereas expression of protein from the wild type gene was not (Fig 2B). The purified protein yield for FMP003 was 70 µg/g of wet cell paste (Fig. 2C, Table 1). No protein could be isolated from bacteria expressing the wild type gene fragment (Fig. 2C, Table 1). The purified FMP003 protein was determined to be soluble based on its presence in the supernatant fraction after centrifugation at 100,000×g for 1 h (data not shown). The details of using the Codon Harmonization Algorithm to generate a recommended sequence for the FMP003 gene are given in Table S1.

**Table 1:** Summary of Protein Expression Levels, Protein Yield, and Synonymous Codon Changes for the Constructs Described in this Study

	aExpression Level		Purified Protein Yield			
	% of total protein		(mg/g cell paste)		Synonymous Codon Changes	
**Protein**	**WT Gene**	**CH Gene**	**WT Gene**	**CH Gene**	**# codons**	**# changes**
FMP003	Not Detected	Not Detected	Not Detected	0.07^b^	354	1
FMP010	Not Detected	∼10%	Not Detected	4^b^	354	244
MSP1_42_-3D7.2	2%	∼10%	0.8 b	3	375	246
MSP1_42_-Camp.2	Not Detected	∼10%	ND	12	373	245
LSA-NRC^H^	Not Detected	∼10%	ND	8^b^	443	288

a Evaluated by densitometry of PAGE Gels Stained with Coomassie Blue R-250

b Purified according to cGMP standards

ND =  not done

WT = wild type

CH = codon harmonized

### Full Gene Codon Harmonization

**Figure 3:**
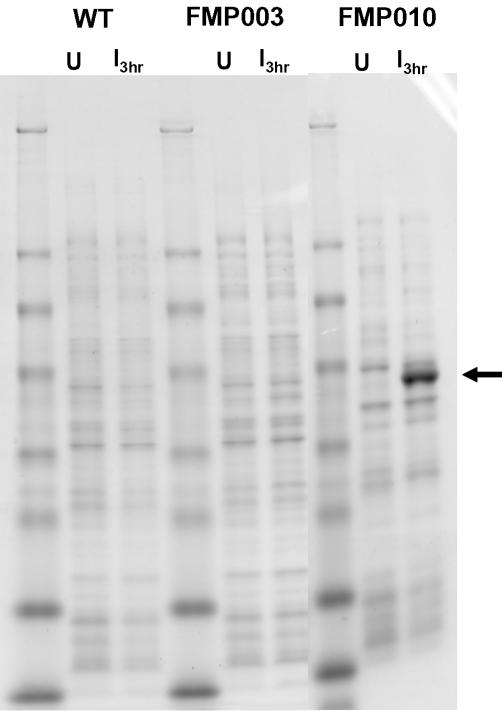
Comparison of expression levels of (WT), FMP003, and FMP010 proteins. Total *E. coli* cell lysates were separated by SDS-PAGE followed by staining with Coomassie Blue at either the time of induction (U) or 3 hours post induction (I_ 3hr_) with 0.1 mM IPTG. The arrow indicates the migration of MSP1_42_ protein.

**Figure 4:**
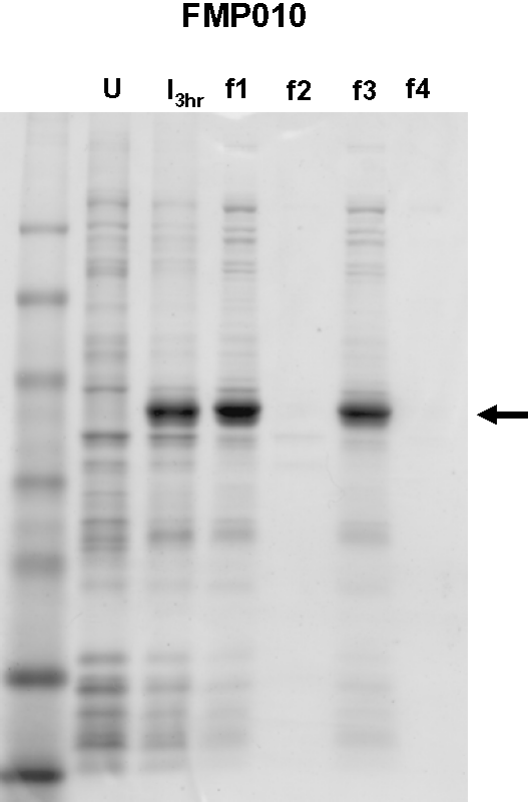
Solubility of the FMP010 protein product. U, lysate from uninduced cells; I_3hr_, lysate prepared 3 hours after induction with 0.1 mM IPTG; f1, 30,000×g supernate from induced cell lysate; f2, 30,000×g pellet from induced cell lysate; f3, 100,000×g supernate of 30,000×g supernate; f4, 100,000×g pellet of 30,000×g supernate,. The arrow indicates the migration of FMP010.

The algorithm was further tested by preparing a “fully harmonized” MSP1_42_ (FVO) gene fragment (designated FMP010), which was completely recoded according to the harmonization approach described in “Materials and Methods.” The value for len/30 was calculated to be 11 and setting the algorithm's reference value for infrequent codon usage in *P. falciparum* to 11% predicted seventeen discrete link/end segments. Setting this reference value to 10.9% predicted only six link/end segments. Densitometry of the gel shown in Fig. 3 shows that after induction with IPTG, MSP1_42_ expression level from FMP010 exceeded levels from wild type sequence by at least 100-fold, and the purified protein yield was 4 mg/g wet cell paste (Table 1). Protein in the cell lysate after microfluidization was fully soluble in the presence of 0.3% sarkosyl; it remained in the supernatant fraction after sequentially centrifuging at 30,000 and 100,000×g for 1 h (Fig. 4, f1 and f3, respectively), and was absent from the corresponding pellets (Fig 4, f2 and f4, respectively). The details of using the Codon Harmonization Algorithm to generate a recommended sequence for the FMP010 gene are given in Table S1.

**Figure 5:**
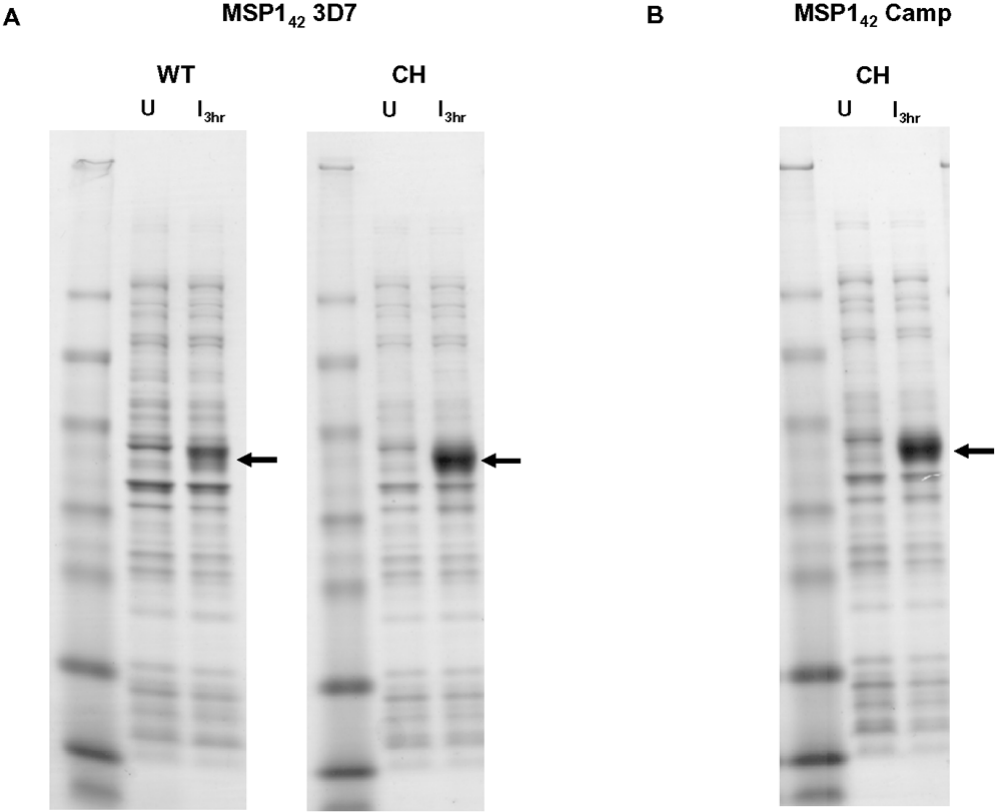
Comparison of expression levels of wild type (WT) and full gene codon harmonized (CH) MSP1_42_ gene fragments from 3D7 and Camp strain *P. falciparum*: U, cell lysates from uninduced cells; I _3hr_, lysate prepared 3 hrs post induction, respectively, after induction with 0.1 mM IPTG. Samples were separated by SDS-PAGE and stained with Coomassie Blue. Panel A; MSP1_42_ (3D7): expression of wild type (WT) and codon harmonized MSP1_42_ 3D7.2 (CH) genes respectively. Panel B; expression of codon harmonized MSP1_42_ Camp.2 gene. The arrows indicate the migration of MSP1_42_.

We applied the same algorithm to the expression of MSP1_42_ (3D7) and MSP1_42_ (Camp), which are alternative alleles to MSP1_42_ FVO. The len/30 values for MSP1_42_ (3D7) was equal to 12, and setting the reference value for infrequent codon usage to 11% predicted seventeen discrete link/end segments. Setting this reference value to 10.9% predicted only six link/end segments. Three hours after IPTG induction, expression levels of codon harmonized MSP1_42_ (3D7) from pET(AT) MSP1_42_ 3D7.2 were much higher than from the native sequence (Fig. 5A, WT and CH, respectively, lanes 3), and product yield increased four-fold, from 0.8 mg/g wet cells for the native sequence to 3.0 mg/g wet cells from the codon harmonized sequence (Fig 5A, Table 1). Basal levels of expression prior to induction are shown in lanes marked U (un-induced cells).

The Camp allele of MSP1_42_ is a natural chimera of the FVO and 3D7 alleles. The FVO and 3D7 alleles have different protein sequences (58% identity) [27] within the N-terminal 300 residues of the protein, known as MSP1_33_, but differ only by four amino acids within the mature C-terminal 96 amino acid fragment known as MSP1_19_ (Q14/ E14, K61/T61, N70/S70, and G71/R71, respectively; numbering relative to the MSP1_19_ fragment). The Camp allele is identical to the 3D7 allele within the MSP1_33_ domain and the N-terminal 80% of MSP1_19_, including E14_,_ but changes to FVO (K61, N70, and G71) for the C-terminal 20% of MSP1_19_. The len/30 values for MSP1_42_ (Camp) was equal to 12, and setting the reference value for infrequent codon usage to 11% predicted nineteen discrete link/end segments, while setting it to 10.9% or less identified only six such regions. Expression levels for codon harmonized MSP1_42_ Camp.2, at 12.0 mg/g wet cells (Fig 5B, Table 1) were very high; we were not able to express wild type MSP1_42_ (Camp) in *E. coli* (Table 1).

**Figure 6:**
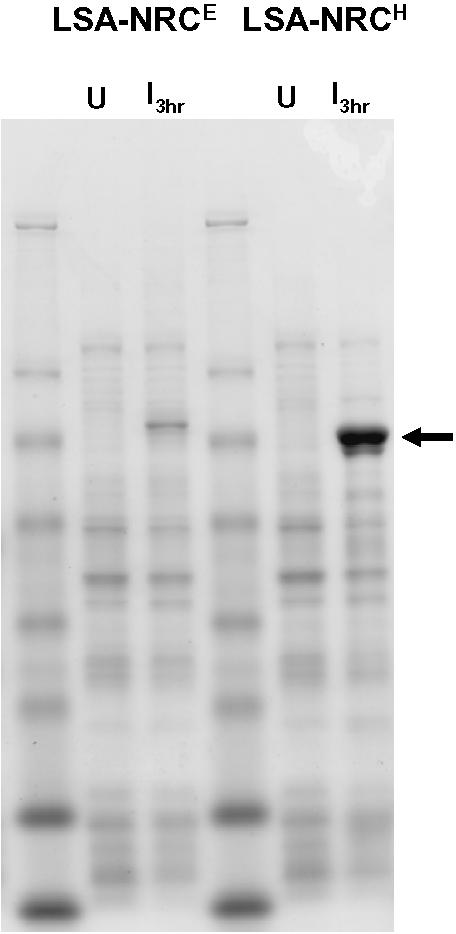
Evaluation of expression levels for the *E. coli* codon optimized LSA-NRC^E^ (3D7) and full gene codon harmonized LSA-NRC^H^ (3D7). Total *E. coli* cell lysates were separated by SDS-PAGE followed by staining with Coomassie Blue at the time of induction (U) and 3 hours post induction (I _3hr_) with 0.1 mM IPTG. The arrow indicates the migration of LSA-NRC.

Expression of the codon harmonized LSA-NRC^H^ malaria vaccine candidate, which has been described elsewhere [26], has proven to be the best approach, to date, for production of this protein in *E. coli*. Attempts to express the native *P. falciparum* sequence for this gene fragment in *E. coli* were not successful owing to plasmid instability (David Lanar, WRAIR, personal communication). The same was also true for the LSA-NRC^E^ gene, a synthetic gene that was “codon optimized” by substituting with codons that are used most frequently in *E. coli* proteins [26]. For this latter construct, plasmid loss was especially problematic during exponential growth phase, suggesting that the expression of the codon optimized protein induced a host cell stress response. On the other hand, the synthetic gene designed by using codon harmonization (LSA-NRC^H^) yielded a dramatic increase in expression levels when compared with LSA-NRC^E^ (Fig. 6, I_3hrs_). The purified protein yield for LSA-NRC^H^ was 8 mg/g wet cell paste (Table 1). The protein produced from this codon harmonized gene was soluble, even in the absence of sarkosyl, and the problem of plasmid loss during exponential growth was resolved. These results further support the hypothesis that the LSA-NRC^E^ and LSA-NRC^H^ protein products fold differently when expressed in *E. coli* and that the expression of LSA-NRC^H^ protein did not induce a deleterious host cell stress response.

## Discussion

The recent findings that genetic polymorphisms reflecting synonymous codon substitutions are not “silent” and are implicated in the development of various disease states mediated through splicing defects refutes the long held dogma that synonymous mutations are neutral [28]–[30]. Even a single synonymous codon substitution within a coding region can lead to proteins with altered substrate specificities [2] or enzymatic activities [1], indicating significant changes in protein structure. Thus, it is possible that subtle modulation of nucleotide sequence may also serve to regulate protein structure and function, and such sequence may have experienced evolutionary pressure to produce fully functional proteins. Clearly, the present findings portend problems in the area of applied biotechnology for heterologous protein expression from distantly related organisms with disparate codon bias. Consequently, we have developed an algorithm that adjusts the tRNA isoacceptor availability found in the natural host to that of the expression host; this may be needed to provide optimal translational kinetics in the expression host.

The basic concepts underlying the “codon harmonization” algorithm are derived from evidence provided in Thanaraj, et al, where codon usage frequency patterns from *E. coli* proteins having known structures were analyzed [22]. They showed empirically that lower frequency codon preferences (rare codons) tend to cluster within the regions of mRNA that encode the link/end segments that separate elements of higher ordered structure . These segments are approximately fifteen residues long and are encoded by clusters of infrequently-used codons [22], [31] that are separated by 1–10 codons [23]. As few as two consecutive infrequently used codons can reduce the steady-state density of ribosomes on mRNA [32]. Slowing ribosomal transit-time through such regions may allow concurrent translation and acquisition of ordered structure by a structural element, and this would be completed prior to the synthesis of the next element.

Studies of the prokaryotic ribosomal tunnel during protein synthesis support its role as an active modulator of nascent peptide secondary structure formation [33]. High-resolution electron micrographs of the 70S ribosome from *E. coli* show that the 50S subunit contains a bifurcating tunnel that is 85Å to 110Å in length as measured from the amino peptidyl transferase center to the exit site on the distal surface [34], [35], which can accommodate nascent peptides of 30 to 72 amino acids, depending on secondary protein structure [7]. The diameter of the tunnel is sufficiently large to accommodate an alpha helix structure. The exit of the tunnel at the ribosomal surface, which is 25–30Å in diameter, appears to accommodate chaperones, such as Trigger Factor [36], [37], which, as necessary [38], can interact with partially folded nascent polypeptides to promote their complete folding. This may serve to shield them from proteolysis prior to complete structure formation as they are extruded from the tunnel [39].

The benefits derived by this rational process of codon substitution are most dramatically shown by our limited mutagenesis approach to create a single targeted synonymous codon replacement for I141 within the sequence encoding a putative link/end segment contained with MSP1_42_ (FVO) protein. Making this single base change, (i.e., to produce the FMP003 protein), increased yields of soluble product to approximately 70 µg protein/g of wet cell paste, this being at least ten-fold over what was achieved with the native sequence. The MSP1_42_ FMP003 antigen was subsequently produced under GMP conditions and shown to be highly immunogenic and efficacious against malaria challenge in an *Aotus* monkey study [25]. However, the yield of FMP003 protein was too low to be of practical use for vaccine development. Therefore we decided to “harmonize” codons throughout the entire gene sequence for MSP1_42_ (FVO), producing FMP010, and obtained a sixty-fold increase in expression over the level that was detected for FMP003.

Expression levels for soluble protein from the codon harmonized MSP1_42_ 3D7.2 and MSP1_42_ Camp.2 genes equaled the levels produced for FMP010. Our successes with the -FVO and -Camp alleles are notable, as we detected no recombinant protein when the native *P. falciparum* gene sequences were used for *E. coli* expression. Thus, this approach has overcome a practical barrier and recombinant proteins for these three genes are currently being evaluated in pre-clinical studies to determine their vaccine potential.

In addition to showing that the FMP003 protein produced a strong malaria protective effect in vaccinated monkeys [25], we observed that FMP010 induced antibodies that inhibit malaria parasite growth *in vitro* at levels comparable with FMP003 (data not shown). Such antibodies are known to be directed to important conformational epitopes in the antigen [40], [41]. Details for the pre-clinical evaluation for FMP010 will be described elsewhere.

The improved expression of soluble protein was not a consequence of simply changing the G/C ratio in the *P. falciparum* target genes. As we show here with the LSA-NRC^E^ gene fragment, codon optimization, or synonymously substituting high frequency codons throughout the gene, for expression in *E. coli* allowed for the production of very little protein. Codon harmonization rectified this problem by preventing plasmid loss during exponential growth, which suggests that the LSA-NRC^E^ expression product induced a deleterious host cell response. The MSP1_42_ proteins from the FVO and 3D7 strains *P. falciparum* have been expressed at high levels in *E. coli* from codon optimized genes, but these proteins were insoluble and required refolding *in vitro* [42], [43].

Codon harmonization appears to offer excellent prospects for design and expression of heterologous proteins, at least in *E. coli*; whether or not it will be useful for other expression hosts remains to be determined. If such adjustments for relative codon usage can improve reliability of functional protein expression, this approach may represent a paradigm shift for heterologous protein expression, with important consequences for both structural biology and biotechnology. However, one may anticipate that control mechanisms other than the availability of tRNA isoacceptor molecules can also affect co-translational folding under different growth conditions or at different stages in the cell cycle. The studies presented here underscore the importance of continuing to achieve general solutions to problems of heterologous protein expression. Advances based on integration of proteomic and genomic analysis may not be fully realized until the target genes and the synthetic potential of the expression organism are completely integrated; failing to achieve such a balance may leave many potential vaccine and biopharmaceutical products undiscovered.

## Materials and Methods

### Algorithm for Codon Harmonization

Design of synthetic genes with this algorithm requires two major steps: identifying and recoding putative link/end segments, followed by the recoding of other areas of the gene. The identification of transcript regions that are likely to be translated slowly requires a means to predict such segments. We first marked residues that were one of the ten amino acids (Tyr, His, Trp, Ile, Leu, Val, Ser, Thr, Pro, and Cys) that appear most frequently in unstructured regions of *E. coli* proteins whose structures are known [20]. Second, we determined the frequency with which every residue's respective codon is used in the native expression host (in this case *P. falciparum*) as a percentage of all isoacceptors that can encode that residue. Third, we selected a reference level that defined low codon usage frequency in the native expression host; initially this reference level was selected to be 15%. Fourth, we marked the residues that both were on the list of ten residues that appear most frequently in unstructured protein regions and were determined to have codon usage rates less that the selected reference level. Fifth, we inspected the results and counted the number of regions that contained discrete clusters of infrequently used codons. The optimal number of discrete clusters was calculated to be equal to the len/30 where len is the number of residues in the protein [20]. Steps 3–5 of this process were reiterated, selecting a lower or higher reference frequency to decrease or increase the sensitivity for optimizing the number of infrequent codon clusters detected. Occasionally we identified codons that were potential sites for translational pausing, but were isolated rather than occurring as part of a cluster. In such a case the *P. falciparum* codon was replaced with the *E. coli* codon that most closely matched its frequency of usage. For this work, the final iteration of the algorithm used an expression host codon frequency reference value of 11%.

After identifying and harmonizing the usage frequencies of critical codons in the putative link/end segments, the rest of the gene is harmonized by selecting synonymous codons from the heterologous expression host having usage frequencies that best reflect the usage frequencies found for the native gene in the native expression host; the selection logic is as follows: preferably equal to, but if not available then the nearest greater than or less than.

### Construction of Expression vector pET(K)

A multi-step cloning strategy was used to generate the final expression vector, pET(K) from a precursor known as pET(AT) [44]. Elimination of *bla* gene expression from pET(AT) was achieved by excising the transcriptional and translational regulatory region with DraI (New England Biolabs, Beverly, MA) and SspI (Roche, Indianapolis, IN) and re-ligating the ends, producing pET(T), which confers tetracycline resistance. The pET(K) was prepared from linearized pET(T), which was prepared by digesting with Tth111I (New England Biolabs), treating with T4 DNA polymerase (Roche) to generate blunt ends and digesting with SapI (Roche). The *kan* gene was isolated from the pZE2.1PAC.4 vector [45] by digesting with AatII (Roche) and SacI (Roche), and a blunt ended insert was prepared by treating with T4 DNA Polymerase (Roche). Restriction mapping of pET(K) showed that the *kan* coding sequence is on the plus strand.

### Design, preparation and cloning of the synthetic gene fragments

**1. Site-directed mutagenesis for FMP003:** The codon harmonization algorithm was applied to the gene fragment encoding MSP1_42_ from the FVO strain of *P. falciparum* **(GenBank accession no. L20092)**. A single synonymous codon change, associated with a predicted link/end segment from the N-terminal portion of the protein (codon 141 (Ile); bp 423 (C->A), was selected for harmonization. Mutagenesis, cloning and expression of this synthetic gene fragment, defined as FMP003 has been described elsewhere [25].

2. **Full codon harmonization of MSP1_42_ FVO gene:** The synthetic gene sequence for the MSP1_42_ FVO synthetic gene fragment or FMP010 (**GenBank Accession no. DQ926900**) was developed by applying the codon harmonization algorithm over the entire gene sequence. For gene synthesis, consecutive pairs of complementary oligonucleotides (each 50–60 bases, having 12–13 bases of unpaired sequence on the 5′ ends) were used to prepare four separate larger segments by using sequential PCR steps. After TA cloning and transformation of One Shot TOP 10 supercompetent cells (Invitrogen, Carlsbad, CA) using ampicillin resistance, transformants were isolated and inserts were sequenced to confirm all codon modifications. Each of the four segments of ∼300 nt contained unique restriction enzyme sites at their termini, which allowed final assembly: fragment 1, 5′ NdeI-3′ HincII; fragment 2, 5′ HincII-3′ BsrG; fragment 3, 5′ BsrG I–3′ BstBI; fragment 4, 5′ BstBI–3′XhoI. The assembled MSP1_42_ fragment (1130 nt) was cloned into the TA vector pCR 2.1 (Invitrogen), producing pCR 2.1-MSP1_42_ FVO, and transformants were isolated and verified by DNA sequencing. The MSP1_42_ FVO insert was excised from pCR 2.1-MSP1_42_ FVO by digesting with NdeI and XhoI, the insert was purified on a 1% agarose gel, and cloned into the final pET(K) expression vector also prepared with NdeI and XhoI (Veritas, Inc., Rockville, MD). Competent host cells (One Shot TOP 10) were transformed to kanamycin resistance, and restriction analysis was used to identify correct clones for DNA sequencing. For expression, the plasmid was used to transform the BL21-DE3 expression host (Novagen, Milwaukee, WI).

**3. Full codon harmonization of the MSP1_42_ 3D7.2 gene fragment:** The sequence for the synthetic MSP1_42_ 3D7.2 gene fragment (**GenBank Accession no. DQ926901**) was developed by applying the full codon harmonization algorithm. The gene was constructed by using PCR amplification in conjunction with proprietary protocols (Retrogen, Inc., San Diego, CA). The amplified DNA was inserted into the pCR-Blunt vector (Invitrogen) creating the vector pCR 2.1-MSP1_42_ 3D7.2. Both strands of the pCR 2.1-MSP1_42_ 3D7.2 insert were verified by sequencing using an ABI 377 automated sequencer, and confirmed to be correct. The insert was excised from the plasmid by digesting with NdeI and NotI, purified on a 1% agarose gel, and subcloned into the final pET(AT) expression vector, which was also prepared by digesting with NdeI and NotI. The plasmid was designated pET(AT) MSP1_42_ 3D7.2. BL21-DE3 transformants were selected on ampicillin, and authenticity of the MSP1_42_ insert within each plasmid was verified by sequencing purified plasmid.

**4. Full codon harmonization of MSP1**_42_ **Camp gene fragment:** The Camp allele of MSP1_42_ is a chimera of the 3D7 and FVO alleles, and was made by splicing AlwNI (New England Biolabs) fragments corresponding to the 5′end and 3′end of the codon harmonized 3D7 and FVO genes, respectively. Thus, pET(AT) MSP1_42_ 3D7.2 digested with Alw NI and dephosphoryalated with shrimp alkaline phosphatase (Roche) provided the 5′ end of the gene, while the plasmid encoding FMP010 was digested with Alw NI provided the 3′ end of the gene. Vector and insert were gel purified using QIAEX II (QIAGEN, Valencia, CA) and ligated using T4 DNA ligase (Roche). Ligations were used to transform electrocompetent B834 DE3 bacterial cells (Novagen) to tetracycline resistance, producing an intermediate clone designated pET(T) MSP1_42_ Camp.2; this cloning eliminated ampicillin resistance from the vector. The final expression plasmid, pET(K) MSP1_42_ Camp.2, was prepared by digesting both the insert and pET(K) vector DNA's with NdeI and NotI, the digested DNA's were gel purified, ligated and transformed into BL21-DE3. Colonies were screened for correct inserts using DNA restriction digestion and analyzed 1% agarose gels stained with ethidium bromide. Authenticity of the MSP1_42_ insert was verified by sequencing purified plasmid (**GenBank Accession no. DQ926902**).

**5. Codon harmonized LSA-NRC^H^ gene fragment.** A codon harmonized synthetic gene was comprised of fragments from the N-terminus (residues 28-154) and the C-terminus (residues 1630-1909) as well as two 17 amino acid repeats of LSA-1 of the *P. falciparum* 3D7 clone **(GeneBank ID no. A45592)**. The molecular construction of the LSA-NRC^H^ synthetic gene **(GenBank Accession ID no. AY751501)** and its expression from the pET(K) expression vector have been described elsewhere [26].

**6. Codon optimized LSA-NRC^E^ gene fragment.** A synthetic gene encoding the same protein sequence as described above for the LSA-NRC^H^ was designed based on using the most abundant codons associated with highly expressed proteins in *E. coli* [46]. The gene was synthesized commercially (Retrogen, San Diego, CA), cloned and verified as described for the MSP1_42_ (3D7) gene fragment above. The synthetic gene was ligated into the NdeI and NotI sites of pET(K) to prepare the expression construct pET(K) LSA-NRC^E^. The recombinant plasmid was amplified by transforming *E. coli* DH5α using kanamycin resistance, and the gene insert was verified by sequencing both DNA strands. For expression of the LSA- NRC^E^ protein, the plasmid was used to transform *E. coli* Tuner (DE3) (Novagen) using kanamycin resistance in the presence of 1% glucose.

### Expression

Shake flasks containing 100 mL of phytone-based super broth and 0.5% glucose (all MSP1_42_ constructions) or Select APS Superbroth medium (Difco, Becton Dickinson, Sparks, MD) and 1% glucose (all LSA1 constructions) were inoculated with 0.1 mL of cryopreserved cells and incubated in a shaking incubator at 175 rpm and 30°C (all MSP1_42_ constructions) or 37°C (all LSA-NRC constructions) overnight for 10–14 hours. Cultures also contained the appropriate antibiotic for the clone (100 µg/ml ampicillin for MSP1_42_ 3D7 and 50 µg/ml kanamycin for MSP1_42_ Camp and MSP1_42_ FVO (FMP010), and 35 µg/ml kanamycin for all LSA-NRC constructions). The overnight cultures were used to inoculate 10 L fermentors filled with the media and antibiotics described above for each clone, and the cultures were fermented at the appropriate temperatures with either 400 or 600 rpm of agitation to an optical density at 600 nm of 4–7 OD (all MSP1_42_ constructions) or 8–10 (all LSA-NRC constructions). In all cases, protein expression was induced for 2–3 hours by adding dioxane-free IPTG (Gold Biolabs, St. Louis, MO) to a final concentration of 0.1 mM. Bacteria were harvested by centrifugation at 4°C, 15,000 rpm and the cell paste was stored frozen at −80±10°C.

### Evaluation of protein expression and immunoblotting

Protein expression was evaluated either by staining with Coomassie Blue R250 (Bio-Rad, Richmond, CA) after SDS-PAGE [47] of whole cell extracts, or by western blotting of partially purified proteins. In either case, proteins were separated under nonreducing conditions. Proteins were electrophoresed with Tris-Glycine buffering (Invitrogen), on 4–20% gradient gels. Whole cell extracts were prepared by extracting 0.5 OD_600_ of cells with SDS-PAGE sample buffer. For protein purifications, frozen cell paste was thawed and cells were lysed by microfluidization (Model M-110Y, Microfluidics Corp. Newton, MA) as described previously [44]. For western blotting, MSP1_42_ proteins were detected by probing with polyclonal rabbit anti-MSP-1_42_ [44]. Western blots were prepared by using nitrocellulose membranes (PROTRAN, Schleicher & Schuell, Inc., Keene, NH) which were subsequently blocked using 5% nonfat dry milk and 0.1% Tween 20 in PBS, pH 7.4. The secondary antibodies were anti-rabbit IgG (Fc) alkaline phosphatase conjugates (Promega, Madison, WI) and reactions were detected with nitro-blue tetrazolium (NBT) and 5-bromo-4-chloro-3-indolyl phosphate (BCIP) (Sigma Chemicals) in 100mM NaCl, 5mM MgCl_2_, 100mM Tris-HCl, pH 9.5. All antibodies were diluted into phosphate buffered saline, pH 7.4 containing 0.1% Tween 20, and the same buffer was used for all washing steps. MSP1_42_ proteins were further evaluated for maintenance of structure by probing western blots with six different conformation-dependant mAbs [41]. These studies required at least partial purification of the expressed proteins by chromatography on Ni^2+^ chelate (Ni-NTA Superflow, QIAGEN, Valencia, CA) in order to allow for epitope stabilization by formation of intra-molecular disulfide bridges within the C-termini of the molecules [25], [44].

## Supporting Information

Table S1 Illustration of Codon Harmonization. Example showing application of the codon harmonization algorithm over a segment of MSP1-42 FVO coding sequence.
(0.04 MB XLS)
